# Recombinant Adeno-Associated Virus Serotype 9 Gene Therapy in Spinal Muscular Atrophy

**DOI:** 10.3389/fneur.2021.726468

**Published:** 2021-10-13

**Authors:** Katarzyna Kotulska, Aviva Fattal-Valevski, Jana Haberlova

**Affiliations:** ^1^Department of Neurology and Epileptology, The Children's Memorial Health Institute, Warsaw, Poland; ^2^Pediatric Neurology Institute, “Dana-Dwek” Children Hospital, Tel Aviv Sourasky Medical Center, Tel Aviv University, Tel Aviv, Israel; ^3^Neuromuscular Center, Department of Pediatric Neurology, Faculty Hospital Motol, 2nd School of Medicine Charles University, Prague, Czechia

**Keywords:** spinal muscular atrophy, spinal muscular atrophy treatment, onasemnogene abeparvovec, gene therapy, AAV9

## Abstract

Spinal muscular atrophy (SMA) is an autosomal recessive neuromuscular disease caused by deletion or mutation of the *SMN1* gene. It is characterized by a progressive loss of motor neurons resulting in muscle weakness. The disease affects 1 in 11,000 live births and before the era of treatment SMA was a leading genetic cause of mortality in infants. Recently, disease modifying therapies have been introduced in clinical practice. They include intrathecal and oral antisense oligonucleotides binding to pre-mRNA of *SMN2* gene and increasing the translation of fully functional SMN protein as well as *SMN1* gene replacement therapy. Onasemnogene abeparvovec uses the adeno-associated virus 9 (AAV9) vector to deliver the *SMN1* gene. Phase 1 and phase 3 clinical trials showed that a single administration of onasemnogene abeparvovec resulted in improvement of motor functions in the majority of infants with SMA. Currently, phase 3 trials in SMA1 and SMA2 patients, as well as presymptomatic infants diagnosed with SMA, are ongoing. The drug was approved for medical use in the US in 2019, and in Japan and the European Union in 2020. Thus, first real-world data on efficacy and safety of onasemnogene abeparvovec in SMA patients are available.

## Spinal Muscular Atrophy: Symptoms, Genetics, Types, Diagnosis, and Natural History

Spinal Muscular Atrophy (SMA) is a devastating autosomal recessive neuromuscular disorder characterized by a progressive loss of spinal motoneurons and leading to muscle weakness and atrophy. It is one of the most common monogenic diseases. In the large study on SMA epidemiology, which was conducted in the USA on a multiethnic group of 68,478 people, SMA incidence was calculated to be 1:11,000 births and a carrier frequency 1 in 47–72 individuals (1). A recently published report from the German newborn screening pilot program indicated an SMA incidence of 1:7,000 (43/297,163) (2). In Israel, the carrier screening program started in 2013 revealed 5,741 cases in 309,352 individuals (carrier rate 1:54) (3). Before the era of treatment, SMA was the leading genetic cause of death in infants and young children.

In its natural history, SMA is characterized by a broad age spectrum of disease onset and course, including the severity of symptoms and signs and subsequent complications (4). In all SMA patients present with muscle weakness and atrophy predominantly visible in the proximal muscles of the shoulder and pelvic girdle, hypo/areflexia, fasciculations in the denervated muscles (for example fasciculations of tongue muscles are frequently seen in infants with SMA) and skeletal deformities, including scoliosis and contractures. Weakness of respiratory muscles may lead to respiratory failure and exposes the affected patients to the increased risk of infections. In younger patients with more severe and acute SMA, failure to thrive is frequent, whereas in older patients there is an increased risk of obesity-associated with limited mobility. SMA patients often require gastrostomy due to feeding problems resulting from impaired swallowing and gastroesophageal dysmotility (5, 6). Sleep disorders and excessive sweating are also common comorbidities (7). The cognitive functions in SMA patients are normal or above the average (8, 9).

Until recently, the classification of SMA was based on the age of symptoms onset and motor milestones achieved. Patients were categorized into four main subtypes of the disease: SMA type 1, 2, 3, or 4 (10). SMA type 1 or Werdnig-Hoffmann disease is a severe and acute form of the disease. First symptoms, including severe hypotonia and muscle weakness, are present in an infant before the age of 6 months, so the patients never sit without support and usually require ventilation support or die before the age of two (11–13). In SMA type 2, patients become symptomatic between the age of 6 and 12 months and are unable to stand or walk without support. This form of the disease is characterized by a subacute progression of symptoms. SMA type 3 is also known as Kugelberg-Welander disease and is characterized by the most chronic and most variable clinical course. Patients with this type of diseases are able to walk until the onset of symptoms. Even though SMA types 2 and 3 are less severe and type 3 does not shorten the life expectancy, in both of them the affected individuals inevitably lose their motor functions over time (14–16). SMA with the first symptoms observed in adults is denoted as type 4 (17). In 2011, SMA type 0 was introduced as a fifth category for extremely severe SMA that manifests prenatally and results in respiratory distress in newborns (18).

However, the introduction of disease-modifying treatment in 2016 and treatment of presymptomatic or early symptomatic patients have changed the natural history of the disease. Thus, the classification of patients according to their age at disease onset and best functional status is becoming less and relevant. Instead, the functional classification of patients into walkers, sitters, and non-sitters is increasingly used both in daily practice and clinical trials (19).

SMA is caused by a homozygous deletion of the *Survival of Motor Neuron* (*SMN1*) gene, which is found in about 95% of patients (20). In 3–4% of SMA patients, the *SMN1* gene point mutations are responsible for the disease (21). *SMN1* gene encodes SMN protein which plays important roles in transcriptional regulation, telomerase regeneration, and cellular trafficking (22, 23). It is located on chromosome 5q (locus 5q13), in a duplicated inverted region. Apart from *SMN1* gene itself, this region contains also a centromeric and identical copy of this gene, called *SMN2* gene. Due to a single nucleotide mutation in exon 7, *SMN2* gene is in majority inefficiently spliced and only 10–15% of its mRNA is generated in a full length. Given that SMA patients do not have *SMN1* gene, their SMN protein level depends only on *SMN2* gene and is severely reduced. However, the number of *SMN2* gene copies in human genome varies between 0 and 8. There is a strong inverse relationship between the number of *SMN2* copies and SMA severity (24–26). Patients with the acute form of SMA usually have 1–2 SMN2 copies of *SMN2* gene, whereas 4 copies are common in SMA type 3.

Considering the molecular and cellular background of the disease, three strategies of disease modification in SMA are currently used: replacement of *SMN1* gene, modulation of *SMN2* gene splicing, and enhancement of the function of remaining muscles.

## Overview of Current Treatment Options in SMA

Recent years brought a breakthrough change in the management and prognosis in SMA (27). Currently, three approved therapies are available for SMA: nusinersen, risdiplam, and onasemnogene abeparvovec. In 2017, European Medicines Agency (EMA) and Federal Drug Administration (FDA) approved nusinersen, the first disease-modifying treatment for all SMA patients regardless of their age, SMA type, the number of *SMN2* gene copies, and functional status. Nusinersen is an antisense oligonucleotide modulating *SMN2* splicing (28, 29). Binding to a specific sequence in the SMN2 pre-mRNA, it promotes generation of full length SMN2 messenger RNA and functional SMN protein (30). It is administered intrathecally with four loading doses of 12 mg given on Day 0, 14, 28, and 63 and followed by maintenance doses every 4 months. The phase III, randomized, double-blind and sham-controlled ENDEAR study showed that nusinersen improved survival, muscle function, and developmental motor milestones in infants with SMA1 (31). Another multicenter, double blind phase III CHERISH trial proved that nusinersen increased the Hammersmith Functional Motor Scale—Expanded score in SMA type 2 children aged between 2 and 12 years (32, 33). Recent NURTURE was conducted in 25 children with presymptomatic SMA who had either two (*n* = 15) or three (*n* = 10) copies of *SMN2* gene. In the interim analysis all participants achieved the ability to sit without support, 92% achieved walking with assistance, and 88% achieved walking independently at a median age of 34 months (34).

Currently, nusinersen is available in many countries and the number of treated patients exceeds 11,000. The real-world data confirm its efficacy and safety in all types of SMA and in all age groups (35–37). However, there are case reports suggesting that after the discontinuation of the medication, the disease progresses similarly to its natural course (38).

In 2021, risdiplam (RO7034067), which is also an *SMN2* mRNA splicing modifier was approved by the FDA and EMA for the treatment of SMA in patients 2 months of age and older, with a clinical diagnosis of type 1, type 2 or type 3 SMA or with one to four *SMN2* copies (39). Risdiplam is administered orally and is currently used in several clinical studies in patients with various types of SMA, including presymptomatic. The open-label phase III FIREFISH study in infants with type 1 SMA showed that risdiplam increased the proportion of infants sitting without support after 12 months of treatment (40, 41). In a double-blind, placebo-controlled SUNFISH study in people aged 2–25 years with SMA types 2 or 3 risdiplam improved the score of Motor Function Measure 32 at 12 months (SUNFISH Study data^1^).

Another oral molecule SMN2 increasing generation of full-length SMN2 mRNA and functional SMN protein by modulating *SMN2* splicing, branaplam (LMI070) is studied in SMA type 1 infants.

There are also other emerging strategies in SMA treatment, including enhancement of the contractility and mass of the remaining muscles. A fast skeletal muscle troponin activator—reldesemtiv (CK-2127107^2^) and a highly specific inhibitor of the activation of latent myostatin—apitegromab (SRK-015^3^) are in clinical trials in later onset SMA patients.

In May 2019, FDA approved the first onasemnogene abeparvovec, the first gene replacement therapy for the treatment of SMA patients with <2 years of age with bi-allelic mutations in the *SMN1* gene, including those who are presymptomatic at diagnosis. In May 2020, onasemnogene abeparvovec was approved by EMA with the indication for the treatment of patients with 5q SMA with a bi-allelic mutation in the *SMN1* gene and a clinical diagnosis of SMA type 1, or patients with 5q SMA with a bi-allelic mutation in the *SMN1* gene and up to 3 copies of the *SMN2* gene. EMA limited the use of the medication for patients who weigh 2.6–21.0 kg.

## What is Onasemnogene Abeparvovec?

Onasemnogene abeparvovec is a gene therapy medicinal product that expresses the human SMN protein (42). In contrast to other therapies approved for SMA treatment, nusinersen, and risdiplam, which alter the splicing of *SMN2* gene, onasemnogene abeparvovec is designed to deliver a full-length functional copy of the human SMN1 gene. It is a non-replicating recombinant adeno-associated virus serotype 9 (AAV9) based vector containing the cDNA of the human SMN gene under the control of the cytomegalovirus enhancer/chicken-β-actin-hybrid promoter (43).

In preclinical study in mice it was shown that AAV9 virus, in contrast to AAV8 and AAV6, crossed the blood brain barrier and efficiently targeted cells of the central nervous system (44). One-day-old wild-type animals received facial vein injections of 4 × 1,011 particles of AAV9 vector that expressed green fluorescent protein under control of the chicken-β-actin hybrid promoter. Robust GFP-expression was found in spinal cord, including 60% of motoneurons, and brain, including the neocortex, hippocampus, and cerebellum. In adult-treated animals, AAV9 injected intravenously efficiently targeted astrocytes but did not achieve widespread direct neuronal targeting.

In the animal model of SMA, AAV9 vector containing SMN gene was injected intravenously on postnatal day 1, 5, or 10. Earliest SMN gene replacement rescued motor function and the animals did not show any signs of denervation. Treatment on postnatal day 5 resulted in partial correction, whereas postnatal day 10 treatment had little effect (45).

In another study, delivery of SMN1 transgene using a AAV9 vector significantly decreased the severity of the heart defect in mice model of SMA (46).

Intrathecal injection of AAV9 carrying the human SMN cDNA in mice and non-human primates using a 10 times lower dose compared to the intravenous injection resulted in a similar motoneuron targeting (47).

However, recent study showed that long-term AAV9-mediated SMN overexpression in mouse model of SMA induced a dose-dependent motor dysfunction associated with neurodegeneration. Overexpression of SMN triggered neuroinflammation and the innate immune response, suggesting that uncontrolled SMN protein expression after gene therapy might be toxic (48).

Clinical trials with onasemnogene abeparvovec in human subjects started after animal studies had shown that systemically delivered self-complementary AAV9 crossed the blood-brain barrier, and achieved high levels of neuronal transduction in SMA murine models (44, 49).

The treatments involve single intravenous administration. The recommended dose is 1.1 × 10^14^ vector genomes per kilogram (vg/kg) of body weight (50). When administered intravenously it crosses the blood-brain barrier, targets the central nervous system neurons at all regions, the alpha motor neurons included. In the neurons, the cDNA does not integrate into the host-cell genome, it forms separate episomes (51). The unique structure of cDNA and their promotors enables rapid and sustained expression of SMN protein independently to the host cell-mediated synthesis. As motoneurons are not replicating cells there is a supposition of long-term therapy effectiveness. Until now, there are already 6 years of clinical data of sustaining the effectiveness in SMA patients (52).

Onasemnogene abeparvovec is historically the first systemic causal therapy for SMA. The intravenous administration brings both advantages and disadvantages. The theoretical advantage is, that it may target the systemic abnormality in SMA patient. As there is already information about abnormalities in autonomic and enteric nervous systems, cardiovascular system, and pancreas in SMA patients, allowing the expression of *SMN* gene in these tissues can be favorable. Moreover, new evidence from preclinical studies indicate that SMN protein is required throughout the lifespan of mice (53), especially in muscles. However, this finding rises the possibility of the disadvantage of a single dose of OA. The dosage of gene therapy is calculated on the basis of patient's weight thus the dose calculated for an infant might not be sufficient for an adult.

The disadvantages of systemic administration of AAV9 vector are the affinity to the other than neuronal tissues, high affinity to hepatic, cardiac and blood cells, which cause potential risk for severe side effects (see: safety paragraph). To reduce the side effects corticosteroid therapy and monitoring of liver function, complete blood count, creatinine, and troponin-I level before and at least 3 month after administration regularly is recommended. It should be considered that this recommended immunomodulatory therapy exposes the patient to extra risks.

### Efficacy of Onasemnogene Abeparvovec

The first clinical study with onasemnogene abeparvovec in SMA patients was initiated in 2014 by Mendell et al. at Nationwide Children's Hospital in Columbus, Ohio (50). The AVXS-101-CL-101 or START study was an open-label, phase 1/2a trial conducted in one center, with two different doses of gene therapy used in two cohorts of patients. Infants aged up to six or 9 months, depending on the cohort, with the clinical diagnosis of SMA type 1, homozygous *SMN1* exon 7 deletion, and 2 copies of *SMN2* gene were included in the study. Key exclusion criteria were active viral infection, use of invasive ventilatory support or non-invasive ventilator support for more than 16 h a day, anti-AAV9 antibody titers >1:50, and single base substitution in *SMN2* gene (c.859G>C in exon 7) (NCT02122952^4^). Patients in cohort 1 received one low dose of gene therapy (6.7 × 10^13^ vector genomes per kilogram) and patients in cohort 2 received one high dose of onasemnogene abeparvovec (2.0 × 10^14^ vector genomes per kilogram). The primary outcome of the AVXS-101-CL-101 study was the determination of the safety of gene therapy. The secondary outcome was the time until death or the need for permanent mechanical ventilation, defined as at least 16 h of respiratory assistance needed continuously for at least 14 days. Exploratory outcomes included the achievement of motor milestones and the change in CHOP INTEND score. Sixteen patients were screened for the study and 15 were included. Three were enrolled in cohort 1 and 12 in cohort 2. The mean age of infants at the day of gene therapy administration was 6.3 months in cohort 1 and 3.4 months in cohort 2. The mean baseline CHOP INTEND score was 16 (range 6–27) in cohort 1 and 28 (range 12–50) in cohort 2. All patients were followed up for at least 20 months. At the end of the follow-up, all patients were alive and did not need mechanical ventilation. All patients had increased CHOP INTEND scores. In cohort 2, the mean CHOP INTEND score increased by 24.6 points, and cohort 1 the mean increase of the score was 7.7 points. Eleven patients in cohort 2 were able to sit unassisted and control the head position, nine could roll over, and 2 were able to walk (50). Importantly, none of these patients were able to achieve any of these milestones prior to gene therapy. At the age of 24 months, children from cohort 2 maintained the clinical improvement and demonstrated a substantial increase in peroneal CMAP peak area values (54). The proportion of patients who were able to safely swallow thin liquids increased from 33 at baseline to 83 at 24 months. The better outcome was associated with the earlier age of gene therapy administration and the baseline functional status of the infant. Children in cohort 2 who had their baseline CHOP INTEND score <20 points and received onasemnogene abeparvovec at the age up to 3 months benefitted more from the therapy (mean increase of 35 points in CHOP INTEND scale) than those who received therapy later (mean increase of 23.3 points), despite that the latter had mean baseline CHOP INTEND score of 26.5 points. Best results were achieved in children who had higher CHOP INTEND scores and who received treatment before the age of 3 months. In this subgroup, infants quickly reached CHOP INTEND score above 60, near the scale maximum (55). However, it is important to note that in total 7 of 15 participants in the START study received not only gene therapy, but also other disease-modifying treatment during the 24-month follow-up (56). On the other hand, nusinersen was initiated after the first analyses published in 2017, and only in 3 patients in cohort 1 and 4 patients in cohort 2. It was not used in two patients who achieved independent walking (56).

Further analysis of the results demonstrated that the best predictors of functional amelioration were age <3 months and high CHOP-INTEND scores at baseline (55). The early dosing/high motor group quickly reached a mean score of 60.3 (scale maximum score 64), from a mean baseline of 44.0 and patients gained the sitting unassisted milestone at the youngest age (mean age: 9.4 months). The early dosing/low motor group demonstrated a mean gain of 35.0 points from a mean baseline of 15.7, whereas the late dosing group had a mean gain of 23.3 from a mean baseline of 26.5. The early dosing/low motor group achieved sitting unassisted earlier than the late dosing group (mean age: 17.0 vs. 22.0 months). This suggests that early treatment is the most important predictor followed by a better motor function at baseline.

In March 2021 new long-term safety and efficacy data from 13 START study patients (all 3 from cohort 1 and 10 from cohort 2) were presented during the 2021 Muscular Dystrophy Association Virtual Clinical and Scientific Conference and then published in JAMA Neurology (52, 57). During the mean 5-year follow up, 2 of 3 patients in cohort 1 and all patients from cohort 2 remained free of permanent ventilation. In cohort 2, five of 10 patients did not require any respiratory support and 1 required it only when ill. Prior to onasemnogene abeparvovec administration, 4 patients in this cohort required non-invasive ventilatory support, and 6 patients did not. No previously achieved motor milestones were lost and 2 patients achieved standing with assistance.

The STR1VE study (AVXS-101-CL-303) was an open-label, multicenter phase III study of onasemnogene abeparvovec in infants with SMA type 1 aged up to 6 months who had up to two copies of *SMN2* gene (58). Patients were eligible if had a swallowing evaluation test and up-to-date vaccinations (palivizumab prophylaxis to prevent respiratory syncytial virus infections was also recommended) prior to infusion. Key exclusion criteria were: use of any disease-modifying therapy for SMA, pulse oximetry <96% saturation at screening, tracheostomy/requirement of non-invasive ventilatory support averaging ≥6 h daily over the 7 days, signs of aspiration/inability to tolerate non-thickened liquids based on a formal swallowing test; active infection, and anti–AAV9 binding antibody titer >1:50 Patients received a one-time intravenous infusion of onasemnogene abeparvovec (1.1 × 10^14^ vg per kg) and were followed-up for 18 months. The primary efficacy outcomes were independent sitting for 30 s or longer (at the 18 months of age) study visit and survival, defined as absence of death or permanent ventilation at age 14 months. Twenty-six patients were enrolled in the study, 4 were excluded (1 died, 1 had a body weight lower than the third percentile, and 2 had signs of aspiration or inability to tolerate non-thickened liquids). Thus, 22 received gene therapy at mean age of 3.7 months. Three subjects discontinued treatment: 1 due to adverse events, 1 died, and 1 withdrew from the study. Out of 19 patients who completed the study, 13 (59%) achieved functional independent sitting for 30 s or longer at the 18 months of age. Twenty out of 22 patients (91%) survived free from permanent ventilation at age 14 months. Ability to thrive and weight consistent with age at 18 months were maintained in 9 (41%) and 14 (64%) patients, respectively. Three patients (14%) used permanent non-oral feeding support at age 18 months or at time of withdrawal from the study. Four other patients received intermittent or transient non-oral feeding support. Twelve (55%) patients had a normal or functional swallow result and the other 7 (32%) could safely swallow consistencies other than thin or very thin liquids at age 18 months. Nineteen (86%) patients achieved at least one motor milestone, including sitting without support ≥30 s in 14 patients and independent walking in 1. Mean increases in CHOP INTEND score from baseline were 6.9 points at 1-month post-dosing, 11.7 points at 3 months, and 14.6 points at 6 months.

The results were compared to untreated infants aged 6 months or younger (*n* = 23) with SMA type 1 from the Pediatric Neuromuscular Clinical Research (PNCR) dataset and onasemnogene abeparvovec showed statistical superiority to the PNCR natural history cohort (58).

Two similar studies called STR1VE-EU (NCT03461289) and STR1VE-AP (NCT03837184) were conducted in Europe and Asia-Pacific region, respectively. The results of STR1VE-EU study were presented at World Muscle Society Conference in October, 2020 (Zolgensma data^5^). In the STR1VE-EU trial, 33 patients were enrolled and 1 was excluded because of the delayed administration of gene therapy (at the age of 181 days). The mean age at baseline was 4.1 months and mean baseline CHOP-INTEND score was 27.6. The follow-up ranged from 1.8 to 15.4 months (mean 10.6 months). At the end of follow-up 31 (96.9%) patients were alive and free of permanent ventilation support. One patient died due to respiratory distress. Fourteen (42.4%) patients did not require ventilatory support at any time during the study. Mean increases in CHOP INTEND score from baseline were 5.9 points at 1-month post-dosing, 10.1 points at 3 months, and 13.3 points at 6 months. Six patients (18.8%) acquired the motor milestone of independent sitting for at least 10 s, but the patients' age at the end of follow-up ranged from 6.9 to 18.6 months, so further observations were needed. Twenty-two (66.7%) patients were free of feeding support at the end of follow-up. The results of the STR1VE-AP trial have not been published so far.

A phase I, open-label, dose comparison clinical study of onasemnogene abeparvovec administered intrathecally (NCT03381729, STRONG study^6^) in SMA type 2 patients was started in 2017 and then suspended, as FDA placed it on clinical hold pending further discussions regarding pre-clinical findings (NCT03381729).

The long-term follow-up data from STR1VE cohort were presented during the 2021 Muscular Dystrophy Association Virtual Clinical and Scientific Conference together with data from STR1VE-EU and STRONG studies (57). The mean pooled cohort of 23 patients who received intravenous gene therapy in either STR1VE or STR1VE-EU studies, the mean follow-up was 2.3 years (range 1.6–3.2). Twenty-one patients did not receive any other SMA therapy, 1 received nusinersen and 1 received risdiplam. None required permanent ventilation, 9 patients had respiratory support (BiPAP or cough assist). Eleven new milestones that were not achieved in the parent study were achieved for four patients.

The long-term data of the STRONG study were presented for 8 patients followed up for 1.8–2.8 tears (mean 2.4 years). Onasemnogene abeparvovec was administered at mean age of 43.5 months. Three subjects received risdiplam after gene therapy and 1 was given nusinersen. Four patients acquired new motor milestones and the highest one was sitting without support, which was achieved without nusinersen or risdiplam support for three patients and for one patient receiving risdiplam.

There is an increasing body of evidence that presymptomatic treatment results in best motor outcomes in SMA patients (59). SPR1NT (NCT03505099) is an ongoing multicenter, open-label, phase 3, single arm, trial aimed to evaluate the safety and efficacy of a onasemnogene abeparvovec in presymptomatic SMA patients under 6 weeks of age who have two to three copies of *SMN2* gene. The results of two cohorts in this study were presented during the 2021 Muscular Dystrophy Association Virtual Clinical and Scientific Conference (60, 61). Key inclusion criteria were presymptomatic genetic diagnosis SMA and 2 or 3 copies of *SMN2* gene, ability to tolerate thin liquids, and CMAP ≥2 mV at baseline. Patients with tracheostomy or current use or requirement of non-invasive ventilatory support at any time/duration, treatment with an investigational/commercial product given for the treatment of SMA and having anti-AAV9 antibody titer >1:50 were not eligible. The primary endpoints in the cohort of patients with two *SMN2* gene copies was independent sitting for ≥30 s at any visit up to 18 months of age. The secondary endpoint in this cohort was survival at age 14 months. Exploratory endpoints included proportion of patients requiring respiratory intervention at 18 months of age, WHO, and Bayley-III motor milestones, and CHOP INTEND score.

The primary endpoint for patients with three copies of the *SMN2* gene was independent standing for ≥3 s at any visit up to 24 months of age. The secondary endpoint in this cohort was independent walking for ≥5 steps at any visit up to 24 months of age. Exploratory endpoints included the percentage of patients requiring respiratory intervention at 24 months of age, WHO and Bayley-III milestones, Bayley-III gross motor, and fine motor total scores.

Twenty-nine patients were enrolled in the SPR1NT trial, 14 with two copies of *SMN2* gene and 15 with three copies of *SMN2* gene. The mean age at baseline was 20.6 (range: 8–34) days and 28.7 (range 9–43) days for patients with two and three copies of *SMN2* gene, respectively. The mean CHOP INTEND score was 46.1 in patients with two copies of *SMN2* gene.

In the cohort of patients with two *SMN2* copies, all 14 infants were alive at the time of their most recent visits at a mean age of 14.8 months. No patient has required ventilatory support of any kind during the study and no received feeding support. The mean increase in CHOP INTEND score from baseline was 3.9 points, 13.0 points, 14.8 points, and 15.7 points at 1-month (*n* = 14), 3 months (*n* = 7), 6 months (*n* = 12), and 9 months (*n* = 7) after dosing, respectively. All patients achieved CHOP INTEND scores of ≥50, and 13 patients (92.9%) achieved a CHOP INTEND score ≥58. All study participants also demonstrated gains on Bayley-III fine and gross motor scales (61).

In the three *SMN2* copies cohort, all 15 infants were alive at the time of their last visits with a median age of 15.2 (3.3–21.1) months. No patient has required ventilatory support of any kind and no patient has required feeding support of any kind. Eight of 15 patients achieved the primary efficacy endpoint of independent standing. The remaining seven patients who had not achieved this milestone were all younger than 16.9 months of age. Six patients achieved independent walking. Thirteen patients achieved independent sitting for at least 30 s, 10 of whom achieved this within the normal developmental window of 9.2 months. The remaining patients who had not achieved this milestone were younger than 9.2 months of age. All patients had Bayley-III gross motor and fine motor subtest scaled scores within the normal range for the age group (60).

Patients from the START, STR1VE, STR1VE-EU, STR1VE-AP, SPRINT, and STRONG studies were offered a long-term follow-up for 15 years after the administration of gene therapy, so further data will likely be available in future.

The clinical trials of onasemnogene abeparvovec are summarized in Table 1.

**Table 1:** Summary of clinical trials on onasemnogene abeparvovec for the treatment of SMA.

**Clinical trial**	**Status**	**Brief description of study design**	**Primary endpoint**	**Study population**	**Literature**
START NCT02122952	Completed	Phase 1, open-label, single-infusion, single-center, dose escalation study of safety, and efficacy of intravenous onasemnogene abeparvovec in SMA type 1	Safety (averse events of Grade 3 or higher)	SMA type 1 patients with 2 copies of *SMN2* gene, aged up to 6/9 months	(50)
STR1VE NCT03837184	Completed	Phase 3, open-label, single-infusion, multicenter study conducted in the US, assessing the efficacy, and safety of intravenous onasemnogene abeparvovec in SMA type 1	Independent sitting for at least 30 s	SMA type 1 patients with 1 or 2 copies of *SMN2* gene	(58)
STR1VE-EU NCT03461289	Completed	Phase 3, open-label, single-infusion, multicenter study conducted in the EU, assessing the efficacy, and safety of intravenous onasemnogene abeparvovec in SMA type 1	Independent sitting for at least 10 s	SMA type 1 patients aged up to 6 months	(57)
STR1VE-AP NCT03837184	Completed	Phase 3, open-label, single-infusion, multicenter study conducted in the Asia-Pacific, assessing the efficacy, and safety of intravenous onasemnogene abeparvovec in SMA type 1	Independent sitting for at least 10 s	SMA type 1 patients aged up to 6 months	Results not published, report:
STRONG NCT	Suspended	Phase 3, open-label, single-infusion, multicenter study conducted in the Asia-Pacific, assessing the efficacy, and safety of intrathecal onasemnogene abeparvovec in SMA type 2	Independent sitting for at least 10 s	SMA type 1 patients with 1 or 2 copies of SMN2 gene	(57)
SPR1NT NCT03505099	Ongoing	Phase 3, open label, single-infusion, multicenter study assessing the efficacy, and safety of intravenous onasemnogene abeparvovec in presymptomatic SMA	Independent sitting for at least 10 s	SMA type 1 patients with 1 or 2 copies of SMN2 gene	(60, 61)
START Long-Term Follow-Up (LTFU) NCT03421977	Ongoing	Phase 3, open label, single-infusion, multicenter study conducted in the Asia-Pacific, assessing the efficacy, and safety of intravenous onasemnogene abeparvovec	Independent sitting for at least 10 s	SMA type 1 patients with 1 or 2 copies of SMN2 gene	(52, 57)
SMART NCT04851873	Initiated, not yet recruiting	Phase 3, open-label, single arm, multicenter study to evaluate the safety, tolerability, and efficacy of IV OAV101 in SMA participants	Safety	SMA patient weighting ≥8.5 and ≤ 21 kg	–

The RESTORE registry is a prospective, multicenter, multinational observational database of SMA patients managed according to usual clinical practice. It is sponsored by Novartis company, manufacturer of OA (62). Recruitment started in September 2018. Patients will be enrolled over a 5-year period and followed for 15 years. As of 31 December, 2019, 43 patients dosed with OA were included (63), but no long-term efficacy data are available.

### Safety of Onasemnogene Abeparvovec

The most concerning reported side effects of onasemnogene abeparvovec are acute liver injury, elevated troponin I and transient thrombocytopenia and recently thrombotic microangiopathy (TMA) as side effect was already reported. In 2021 Novartis in agreement with the EMA issued a communication for healthcare professionals on the risk of TMA after onasemnogene abeparvovec (Zolgensma: risk of thrombotic microangiopathy^7^).

In the first clinical trial, the START trial, prednisolone was not included in the protocol. However, after the first patient had had increased liver enzymes 16 times more than normal, protocol amendments stipulated the administration of prednisone 1 mg/kg per day 24 h prior to gene delivery and as a safety measure, a reduced viral load for high dose from 3.3 × 10^14^ vg/kg to 2.0 × 10^14^ vg/kg (50).

In the STR1VE trial, reportedly all patients who received onasemnogene abeparvovec had at least one adverse event (most common was pyrexia). The most frequently reported serious adverse events were bronchiolitis, pneumonia, respiratory distress, and respiratory syncytial virus bronchiolitis, a complication which could be expected in patients with SMA. Three patients experienced serious adverse events reported as drug-related, two of whom with elevated hepatic aminotransferases, and one with hydrocephalus (58).

A recent review on hepatotoxicity following onasemnogene abeparvovec administration analyzed data on 325 patients with SMA who had received onasemnogene abeparvovec in 5 clinical trials, a managed access program (MAP), and a long-term registry (RESTORE), and through commercial use. Of 100 patients included in the analysis with a mean age for therapeutic dosing was 2.9 months, 99 experienced at least 1 adverse event after dosing. The most frequently reported events included pyrexia (50), vomiting (42), hepatic enzyme increased (24), elevated AST (23), elevated ALT (19), elevated liver function (17), decreased platelet count (16), elevated troponin (7), thrombocytopenia (7), cough (7), nasopharyngitis (7), and weight loss (6). Forty-eight patients experienced serious adverse events, of which 11 were considered by the investigator to have been related to onasemnogene abeparvovec. Across all studies, the most frequently reported treatment-related adverse events and serious adverse events were elevations in serum aminotransferase enzyme concentrations and vomiting. In all the studies, the duration of prednisolone treatment was in average 83 days (range: 33–229), with a majority of patients receiving prednisolone for 60–120 days (63).

Subacute liver failure following gene replacement therapy was described in two patients who presented with liver failure and active inflammation on liver biopsy within 8 weeks of receiving onasemnogene abeparvovec (64). Both patients were treated with intravenous methylprednisolone 20 mg/kg/day and were discharged from the hospital within 2 weeks of initial diagnosis. Corticosteroids were weaned off over the next 3.5 months with sustained normalization.

Based on the data analysis, the authors recommended patient evaluation for existing chronic liver disease or predisposition to liver disease prior to gene therapy and liver function monitoring post-gene therapy every 1–2 weeks in the first 2 months after onasemnogene abeparvovec therapy, with dose escalation of corticosteroids as needed for severe hepatitis or acute liver failure. If there is no response to high-dose corticosteroids, then immunosuppressant treatment should be considered [e.g., tacrolimus, sirolimus, everolimus, or antithymocyte globulin; (64)].

Cardiac toxicity was observed in animal studies and transient increase in cardiac troponin-I levels was reported in clinical trials. In the START trial, 8 of 15 patients (53.3%) had elevations in cardiac troponin levels of potential clinical significance (>0.05 μg/L) (50). Of these, 2 patients had had elevated troponin levels prior to administration of onasemnogene abeparvovec. There was one death from a cardiac event 3 days after onasemnogene abeparvovec therapy in Early Access Program. Even though the death was not attributed to onasemnogene abeparvovec as this patient had advanced SMA and multiple medical co-morbidities, a contributory role of onasemnogene abeparvovec cannot be fully excluded. Minor elevations in cardiac troponin I levels were observed in patients, particularly during the first 2 months post-treatment. None of the elevations in cardiac troponin I observed during the clinical trial were considered clinically significant by the investigator. By the end of the study all values had either returned to within the normal range or no longer met the pre-defined criterion for clinical significance. It was suggested that the concurrent use of prednisolone has a protective effect on both liver and heart, thereby preventing life-threatening conditions (inflammation) as seen in mice. It is recommended to monitor troponin-I before onasemnogene abeparvovec infusion and on a regular basis (once a week) for at least 3 months afterwards (65).

Transient decreases in platelet counts were observed after onasemnogene abeparvovec treatment. Platelets count monitoring is recommended prior to onasemnogene abeparvovec infusion and on a regular basis (once a week) for at least 3 months afterwards.

TMA has recently been reported in a few SMA patients treated with onasemnogene abeparvovec, particularly in the first weeks following treatment (66, 67). It is assumed to occur due to dysregulation and/or excessive activation of the alternative complement pathway by genetic or acquired etiology. TMA is treatable in the majority of cases and can resolve with timely and proper interventions.

TMA was first reported in a 6 month-old girl with SMA type 1, intubated and mechanically ventilated due to respiratory failure for ~2 weeks prior to gene therapy (66). Five days post-injection she developed hypertension (140–150 s/80–90 s mmHg), hematuria, proteinuria, acute kidney injury (blood urea nitrogen 52, Cr 0.6), hemolytic anemia (schistocytes on peripheral smear, elevated reticulocyte count of 7.5%, hemoglobin 6.5 and elevated lactate dehydrogenase 4,208) and thrombocytopenia (platelet count <30,000) all consistent with TMA. The patient was treated with antihypertensives, platelet, and red blood cells infusions as well as plasmapheresis. TMA resolved and the child became normotensive on a single antihypertensive. Another 3 children were reported to have developed TMA ~1 week following onasemnogene abeparvovec infusion: 2 of them experienced vomiting, 2 had been previously treated with nusinersen, and 2 had infections with encapsulated organisms. All 3 patients recovered from TMA, one of them with supportive measures alone and the other 2 with additional interventions such as plasmapheresis, increased corticosteroids, and/or transfusions (67).

Up to present, five confirmed cases of TMA in patients aged 4–23 months have so far been reported after treatment with onasemnogene abeparvovec [Zolgensma: risk of thrombotic microangiopathy (see text footnote 7)], among ~800 treated SMA patients. In these five cases, TMA developed within 6–11 days after onasemnogene abeparvovec infusion. The presenting features included vomiting, hypertension, oliguria/anuria, and/or edema. Laboratory data revealed thrombocytopenia, elevated serum creatinine, proteinuria and/or haematuria, and hemolytic anemia (decreased hemoglobin with schistocytosis on peripheral blood smear). Two of the patients also had infections, and both had recently (within 2–3 weeks after onasemnogene abeparvovec administration) been vaccinated. Thus, patients may be at increased risk if they have concurrent infections or other immune triggers. In the acute phase, all patients responded well to medical interventions including plasmapheresis, systemic corticosteroids, transfusions and supportive care. Two patients underwent renal replacement therapy (hemodialysis or hemofiltration). One patient who required renal replacement therapy (hemofiltration) died 6 weeks after the event.

Recommendations are that creatinine and complete blood count are required before onasemnogene abeparvovec infusion. Platelet count should be closely monitored in the week following the infusion and on a regular basis afterward. In case of thrombocytopenia, testing for hemolytic anemia and renal dysfunction should be undertaken. If patients exhibit signs, symptoms, or laboratory findings suggestive of TMA, direct specialist and multidisciplinary advice should be sought and TMA should be immediately managed as clinically indicated. Caregivers should be informed about signs and symptoms of TMA (e.g., bruises, seizures, oliguria) and should be advised to seek urgent medical care if such symptoms occur [Zolgensma: risk of thrombotic microangiopathy (see text footnote 7)].

Long-term safety data of 13 infants who received onasemnogene abeparvovec in the START trial have recently been published. The median age at data cut-off was 38.9 [range, 25.4–48.0] (60) months. Serious adverse events were reported in 8 patients (62%) and included acute respiratory failure (*n* = 4 [31%]), pneumonia (*n* = 4 [31%]), dehydration (*n* = 3 [23%]), respiratory distress (*n* = 2 [15%]), and bronchiolitis (*n* = 2 [15%]). None of them resulted in study discontinuation or death. All 10 patients in the therapeutic-dose cohort remained alive and without the need for permanent ventilation (52).

In the RESTORE registry, adverse events were reported in 28 out of 43 (65.1%) patients who received OA and serious adverse events were reported in 13 patients (30.2%) (63). Most frequent treatment-related adverse events included increased ALT and/or AST levels [in 10 patients (23.3%)]; thrombocytopenia in 4 patients (9.3%); and vomiting in 2 cases (4.7%). The transaminase abnormalities resolved completely in all cases. In 2 children (4.7%) bilirubin elevation and severe elevations in ALT and AST (>40× ULN) were reported. Both children responded to prednisone therapy and could be withdrawn from immunosuppressive therapy.

### Combination Therapy With Onasemnogene Abeparvovec

Even the early treated patients are not always free of all symptoms of the disease and many families seek combination therapy. Given that the 3 approved medications for SMA differ in the mechanism of action, such approach may be appropriate. Recent preclinical studies in mice indicate that administration of two different therapies increasing SMN protein levels might be much more effective in mice with SMA, even when given late symptomatically (68). However, little is known about the efficacy and safety of the combination therapy including gene therapy; moreover, it is usually not available due to economic reasons.

In the START trial, 7 of the 13 patients were receiving concomitant nusinersen during 5 years following the administration of gene therapy. All of them improved in their functional status. No safety issues associated with this combination were reported, however, it is difficult to separate the benefits from gene therapy itself and nusinersen.

Few reports from real world indicate that combination of OA with nusinersen (69, 70) or risdiplam (71) is safe and might bring some additional benefits for the patients who did not respond optimally to gene therapy alone. In few countries such additional therapy is available and reimbursed in patients meeting the criteria of lack of efficacy developed locally. In many other countries, for example in Poland, families try to collect money for gene therapy from public fundraising in order to combine OA with reimbursed treatment with nusinersen. In such situations, it is very difficult to define the benefits and adverse events from both therapies. More importantly, there is a risk that the medical professionals might be exposed to pressure from the side of the family to administer the gene therapy in patients who do not meet the criteria of the maximum weight or minimal functional status.

In future, the definition of non-responder and timing of functional assessments after administration of gene therapy in SMA should be established globally.

### Gene Therapy With Onasemnogene Abeparvovec and Vaccinations

Several vaccines are recommended during early infancy, including live vaccine BCG which is mandatory in some countries (including Poland, Russia, and Brazil). Given that gene therapy is associated with significant immune response and with steroid therapy for at least 1 month, the administration of gene therapy should take the vaccine scheduling into consideration. Specific recommendations are urgently needed.

## Conclusions

In this review, we have discussed the available data on the first gene therapy approved in SMA. We have also identified a number of knowledge gaps relevant to efficacy and safety of OA and its use in the clinical practice.

Until very recently, SMA was considered incurable and fatal. A few years ago, the first disease-modifying therapy was approved and currently, there are three of them, including gene therapy. Despite extremely high costs, these highly innovative therapies have been granted reimbursement in several countries. Together with the implementation of newborn screening programs and treatment of patients in the presymptomatic phase of the disease, they may radically change the prognosis in SMA. However, the history of gene therapy in SMA is relatively short and little is known about its safety and efficacy in a life-long perspective. Long-term efficacy and safety data are urgently needed, as indicated not only by the regulators but also by many experts (72). Efforts should be made to identify widely accepted and feasible timing as well as outcome measures for long-term follow-up of gene therapy recipients and to develop global registries. The criteria of suboptimal response or lack of efficacy should be established for the patients who might benefit from other therapies. On the other hand, it might be difficult to convince the patients who received the single-dose drug and who are doing well to visit the hospital and report their status on a regular basis.

## Author Contributions

All authors listed have made a substantial, direct and intellectual contribution to the work, and approved it for publication.

## Funding

This work was supported by Novartis grant for publishing fee.

## Conflict of Interest

KK, AF-V, and JB receive grant support from Novartis, Biogen, and Roche. The authors declare that this study received funding from Novartis. The funder had the following involvement in the study: grant for publication charge. The funder was not involved in the study design, collection, analysis, interpretation of data, the writing of this article, or the decision to submit it for publication.

## Publisher's Note

All claims expressed in this article are solely those of the authors and do not necessarily represent those of their affiliated organizations, or those of the publisher, the editors and the reviewers. Any product that may be evaluated in this article, or claim that may be made by its manufacturer, is not guaranteed or endorsed by the publisher.

## Footnotes

^1^*New Two-Year Data Show Roche's Evrysdi (Risdiplam) Continues to Demonstrate Improvement or Maintenance of Motor Function in People Aged 2-25 with Type 2 or Type 3 Spinal Muscular Atrophy (SMA)*. Available online at: https://www.roche.com/media/releases/med-cor-2021-03-16.htm (accessed May 15, 2021).

^2^*A Study of CK-2127107 in Patients with Spinal Muscular Atrophy*. Identifier: CT02644668. Available online at: https://clinicaltrials.gov/ct2/show/NCT02644668 (accessed April 3, 2021).

^3^*Apitegromab for Spinal Muscular Atrophy*. Available online at: https://scholarrock.com/our-pipeline/spinal-muscular-atrophy/ (accessed April 3, 2021).

^4^*Gene Transfer Clinical Trial for Spinal Muscular Atrophy Type 1*. Available online at: https://clinicaltrials.gov/ct2/show/NCT02122952 (accessed April 10, 2021).

^5^*Zolgensma*^®^ *Data Including Patients with More Severe SMA at Baseline Further Demonstrate Therapeutic Benefit, Including Prolonged Event-Free Survival, Increased Motor Function and Milestone Achievement*. Available online at: https://www.novartis.com/news/media-releases/zolgensma-data-including-patients-more-severe-sma-baseline-further-demonstrate-therapeutic-benefit-including-prolonged-event-free-survival-increased-motor (accessed May 10, 2021).

^6^*Study of Intrathecal Administration of Onasemnogene Abeparvovec-xioi for Spinal Muscular Atrophy (STRONG)*. Available online at: https://clinicaltrials.gov/ct2/show/NCT03381729 (accessed April 22, 2021).

^7^https://www.ema.europa.eu/en/documents/dhpc/direct-healthcare-professional-communication-dhpc-zolgensma-onasemnogene-abeparvovec-risk-thrombotic_en.pdf (accessed April 10, 2021).
